# Periscope

**Published:** 1881-09

**Authors:** 


### PERISCOPE.

### United States Medical Investigator, April and May.

"M. D." gives accidental proving of *Usnea Barbata*, a kind of lichen :—*Severe headache over entire head or front of head, with a feeling as though the temples would burst, or the eyes would burst out of their sockets.* The same authority reports several cures of headache with this substance.

### Hahnemannian Monthly, April, May and June.

Dr. Thomas reports four clinical cases. In the first one, a case of pneumonia, there was a *sharp, stitching pain in right lung, with dread of motion.* The *slightest motion,* even in turning the head, or lifting the hand, *made him worse.* *Bryonia* was given, and ultimately cured.

The doctor, however, made two mistakes; the first, in continuing the remedy so long as to give the system the additional task of fighting the drug as well as the disease. The second mistake was in changing to *Sulphur*, because of the seeming slowness of action of the *Bryonia*, when the delay was really owing to the excess of the remedy. The doctor, however, quickly saw the error, and corrected it by again giving *Bryonia*. In the light of these two mistakes, this is a very instructive case. The second case of same disease was treated at first with *Aconite*, and then *Bell.* But neither of these remedies gave as good result as *Sulphur*, which was continued until the patient got well. The indications in this case are not very clear. The third case (again pneumonia) was treated with *Bell.*, and then *Rhus tox.* We think *Aconite* should have been given. However, the patient got well, with the exception of a thin, copious, semi-purulent otorrhœa, for which *Hepar* was prescribed with good result. A diarrhœa, in the course of phthisis, was much benefited by "Coto-bark." No indications are known.

Dr. Bayne, of Barbadoes, reports two cases of tetanus, cured with *Passiflora incarnata*, empirically, after the usual homœopathic remedies had been given without effect. This drug has not been proved, hence no indications could be given.

Dr. Bigler gives a condensed translation of Prof. Jæger's Neural Analysis, together with a drawing of the instrument and copies of the "osmograms," or "neural-analytic curves." The condensation is done with very good judgment. We observe, however, that *ozon* in the German has been rendered "oxygen." This is an error; it should be *ozone.*

A partisan of the Milwaukee Academy takes Dr. Farrington to task for his review of Dr. Potter's book. His remarks are interspersed with expressions of "surprise and pity." Dr. Farrington's just criticism seems to have excited the liveliest feelings of apprehension (as to sales) among the friends of the book, so they rush into print in defense of it. Dr. Farrington replies in the same number of the journal. One of his sentences contains the whole truth in a nutshell. He says: "The ostensible object is indeed as asserted by my critic; but under all this is an ulterior purpose, which comes to light in a critical examination of the book. * * * I mean the minifying of Hahnemannism, and the exaggeration of similitudes between the schools *which may point to a distant unification of the two.*" The doctor might have added that we now know the meaning of the terrific and sudden onslaught made upon the pure Hahnemannians a couple of years ago. This attack, so sudden and so violent, was a phenomenon that puzzled us all until at length this book appeared, and then it dawned upon our senses that the object was to advertise the author and thus secure a market for his wares.

Dr. Farrington's concluding sentences are as follows: "Homœopathic cloth though not yet perfect in contexture, is woven systematically, durably and handsomely. Disgusted with the shabby and untrustworthy work of his day, Hahnemann constructed a new loom. The several parts of this masterpiece required untiring labor and consummate skill in their production. They were not the consequences of his seclusion and ostracism. They were the fruits of his genius—a genius which rejected the incompetent apparatus of his day, even before he yet knew of a substitute. If rude hands mar this work, the resulting cloth will show the tampering. Still 'liberty of opinion' is the watchword, and he is to be pitied who clings to the old machine, the Organon, even, I suppose, though not one of its appurtenances, has been proved ineffective, and nothing new offered to equal it, much less supersede it."

The June number contains the report of a discussion on Tonsillitis in the Homœopathic Medical Society of Allegheny County. The paper read gave three varieties of tonsillitis. They are: inflammation of structures around the gland, inflammation of the gland itself, and inflammation of the secreting membrane lining the follicles of the gland. The general tone of the paper and of the discussion was that but little could be done to avert suppuration, and where it seemed to be prevented, the case was not a true inflammation of the gland but only of the follicles, which kind never goes on to suppuration; so that you were deceived about the success of your remedies, don't you see? Nevertheless something is always tried. Each doctor relates his experience—generally empirical—which, of course, does not agree with any other. Thus we have a jumble of opinions instead of an attempt to study out the specific symptoms of the individual patient and an *orderly* application of the indicated remedy singly and in the smallest dose necessary to cure. Indeed, the proceedings of this meeting read more like the discussions in an old school society.

Dr. Swift relates an interesting case of scarlatina, with delayed eruption, successfully treated with the single remedy. *Bell.*, then *Calc-c.*, then *Merc.*, were the remedies used.

Dr. Street successfully reduced a strangulated hernia by *taxis only*. An interesting case.

Dr. Fincké, acting upon the idea of Prof. Jæger, describes a new method of taking observations of the effect of potentized medicines upon the system, in a paper entitled, "The Electro-motorial Test for High Potencies." His method is to connect a minute galvanic battery with a galvanometer. Making the body of the observer a part of the circuit, he takes observations of the effect of the current upon the needle after passing through the human system. He finds that the conduction of the electric current varies according as the nervous system is affected by different drugs. He uses a peculiarly arranged galvanometer. In an astatic combination *each* needle is inclosed in its own coil. Nothing is said as to the connection of these coils with one another; or as to the relation of the resulting rotation in one coil to that in the other. We can only *infer*, therefore, that this arrangement insures greater sensitiveness to electrical influences. The doctor gets some extraordinary readings from his needle. He gets a swing amounting to 245°! When an electric current passes parallel to a magnetic needle, the needle rotates until the poles are at the greatest possible distance from the wire; this of course is 90 degrees. Dr. Fincké, on the other hand, talks about angles of 245°! Something must be out of adjustment in his instrument, else he would never get such an apparent result. Moreover, an astatic combination is diamagnetic—pointing east and west (or nearly so in those combinations unequally magnetized for purposes of investigation). But Dr. Fincké's needles point 45° north of east. These errors should be corrected to render his observations reliable.

### The Clinique, January to June.

In some discussions before the Clinical Society, Dr. Burt declares that milk is a prophylactic in Scarlet Fever. He instances the fact, that children nursing are exempt from the disease; he also relates some instances in his own experience.

Dr. T. S. Hoyne relates three or four interesting cases: One of these was a case of hæmorrhoids, hemorrhage, syphilis, dyspepsia, palpitation of heart and insanity. All conditions were relieved by *Phos.* 30 and 200. A case of Herpes Zoster with the following symptoms: Violent lancinating pains in the left leg, worse in the evening and from touching the part; violent itching of affected parts; burning of parts; was treated with *Zinc.* 30. A case of impetigo was cured with *Graphites* 12 and 200. Prurigo in an old man was cured with *Ignatia* 30 and 200. The indications were: *Sensation as of stinging like bee-stings. It is better in cold weather; disappearance of itching after scratching.*

Dr. Small gives a clinical lecture on malarial fevers. He claims that a great variety of general complaints attended with diarrhœa, fever, sore throat, absence of appetite, weariness, dull headache, etc., are due to the influence upon the system of malarial fever caused by defective sewerage, poisoned water, etc. He illustrates his position by many cases treated homœopathically. Whilst not impugning the truth of Dr. Small's observations, it should be noticed that the old school apply the term *malaria* to every derangement of the system that shows any periodic tendencies whatever. It forms a convenient explanation of everything they don't understand and a sufficient excuse for the prescription of the ever-ready Quinine. Thus when a young lady gets cold in the back of the neck from the drafts of a car window on a cold wet day, and has stiffness of the neck in consequence, it is attributed by her old-school attendant to *malaria*, and Quinine is given in large doses. When it fails to cure, because not it but *Lachesis* was really the remedy, the doctor says the malaria is "stubborn" and "deep seated" and that it will require a long time "to get it out." Hence she is kept in the house all winter—notwithstanding the most tempting invitations, etc., socially. Hence we must be careful how we use the new view given us of indefinable complaints lest we deceive ourselves and our patients, and eclectics make it the pretext for administering massive doses of Quinine in order to save themselves the labor of studying the remedy.

